# Adverse event profile of memantine and donepezil combination therapy: a real-world pharmacovigilance analysis based on FDA adverse event reporting system (FAERS) data from 2004 to 2023

**DOI:** 10.3389/fphar.2024.1439115

**Published:** 2024-07-17

**Authors:** Yihan Yang, Sheng Wei, Huan Tian, Jing Cheng, Yue Zhong, Xiaoling Zhong, Dunbing Huang, Cai Jiang, Xiaohua Ke

**Affiliations:** ^1^ The Institution of Rehabilitation Industry, Fujian University of Traditional Chinese Medicine, Fuzhou, China; ^2^ Department of General Practice, The Second Affiliated Hospital of Wannan Medical College, Anhui, China; ^3^ Department of Rehabilitation Medicine, Shanghai Fourth People’s Hospital, School of Medicine, Tongji University, Shanghai, China; ^4^ School of Health and Rehabilitation, Chengdu University of Traditional Chinese Medicine, Chengdu, China; ^5^ The First Clinical Medical College, Fujian University of Traditional Chinese Medicine, Fuzhou, China; ^6^ Guangdong Provincial Hospital of Chinese Medicine, The Second Clinical School of Guangzhou University of Chinese Medicine, Guangzhou, China; ^7^ Rehabilitation Medicine Center, Fujian Provincial Hospital, Fuzhou, China; ^8^ Shengli Clinical Medical College of Fujian Medical University, Fuzhou, China; ^9^ Fuzhou University Affiliated Provincial Hospital, Fuzhou, China

**Keywords:** real-world pharmacovigilance analysis, FAERS, pharmacovigilance, donepezil, memantine

## Abstract

**Background:**

Donepezil in combination with memantine is a widely used clinical therapy for moderate to severe dementia. However, real-world population data on the long-term safety of donepezil in combination with memantine are incomplete and variable. Therefore, the aim of this study was to analyze the adverse events (AEs) of donepezil in combination with memantine according to US Food and Drug Administration Adverse Event Reporting System (FAERS) data to provide evidence for the safety monitoring of this therapy.

**Methods:**

We retrospectively analyzed reports of AEs associated with the combination of donepezil and memantine from 2004 to 2023 extracted from the FAERS database. Whether there was a significant association between donepezil and memantine combination therapy and AEs was assessed using four disproportionality analysis methods, namely, the reporting odds ratio, proportional reporting ratio, Bayesian confidence propagation neural network, and multi-item gamma Poisson shrinker methods. To further investigate potential safety issues, we also analyzed differences and similarities in the time of onset and incidence of AEs stratified by sex and differences and similarities in the incidence of AEs stratified by age.

**Results:**

Of the 2,400 adverse drug reaction (ADR) reports in which the combination of donepezil and memantine was the primary suspected drug, most of the affected patients were female (54.96%) and older than 65 years of age (79.08%). We identified 22 different system organ classes covering 100 AEs, including some common AEs such as dizziness and electrocardiogram PR prolongation; fall, pleurothotonus and myoclonus were AEs that were not listed on the drug label. Moreover, we obtained 88 reports of AEs in men and 100 reports of AEs in women; somnolence was a common AE in both men and women and was more common in women, whereas pleurothotonus was a more common AE in men. In addition, we analyzed 12 AEs in patients younger than 18 years, 16 in patients between 18 and 65 years, and 113 in patients older than 65 years. The three age groups had distinctive AEs, but lethargy was the common AE among all age groups. Finally, the median time to AE onset was 19 days in all cases. In both men and women, most AEs occurred within a month of starting donepezil plus memantine, but some continued after a year of treatment.

**Conclusion:**

Our study identified potential and new AEs of donepezil in combination with memantine; some of these AEs were the same as in the specification, and some of the AE signals were not shown in the specification. In addition, there were sex and age differences in some of the AEs. Therefore, our findings may provide valuable insights for further studies on the safety of donepezil and memantine combination therapy, which are expected to contribute to the safe use of this therapy in clinical practice.

## 1 Introduction

Dementia comprises a group of brain disorders characterized by acquired behavioral and cognitive deficits, particularly deficits in memory, communication and language; concentration and attention; reasoning and judgment; and visual perception (Maloney and Lahiri, 2016). Dementia is estimated to affect 7% of the global population older than 65, with prevalence rates reaching 8%–10% in developed countries as life expectancy increases (Malik et al., 2022). Studies have shown that spending on dementia will reach $1.6 trillion by 2050, accounting for 11% of all projected healthcare spending (Velandia et al., 2022), imposing significant healthcare costs on society and a heavy burden on families and decreasing the quality of life of affected individuals. Alzheimer’s disease (AD), the most common form of dementia, is one of the top seven causes of death in the United States (Alzheimer’s Disease Facts and Figures, 2024), and according to the Alzheimer’s Association, more than 50 million people worldwide are living with AD, and the number of patients will triple to 152 million by 2050 (Liu N. et al., 2024). There is currently no cure for AD, but medications can be used to slow the decline in cognitive function (Peng et al., 2023). Currently, donepezil and an N-methyl-D-aspartate receptor (NMDA) antagonist (memantine) are approved by the US Food and Drug Administration (FDA) for use in combination as a treatment for AD (Yaghmaei et al., 2023).

Acetylcholine (ACh) is a neurotransmitter that plays an important role in memory and learning (Zannone et al., 2018). Reduced ACh synthesis and the degeneration of cholinergic neurons are the main causes of AD (Gallrein et al., 2023), while beta-amyloid, tau protein accumulation and chronic inflammation are a key feature of AD (Alzheimer’s Disease Facts and Figures, 2024). Studies have shown that donepezil is widely used to treat moderate to severe AD because of its high activity, selectivity, low dose onset of action, and low toxicity (Sharma, 2019). Donepezil leads to improvements in the cognitive symptoms of AD by increasing the availability of ACh through the inhibition of the enzyme acetylcholinesterase and increasing cholinergic transmission (Diaz-Galvan et al., 2023). Evidence from a comprehensive Cochrane review showed improved cognitive function, activities of daily living, and clinical status of patients with mild, moderate, or severe dementia due to AD treated with donepezil for 12 or 24 weeks (Birks and Harvey, 2018). On the other hand, glutamate, one of the major neurotransmitters involved in excitatory pathways, plays an important role in cortical and hippocampal pathways through NMDA receptors (Nisar et al., 2022). NMDA receptor activation allows the influx of calcium ions into postsynaptic neurons and triggers the activation of a pathway important for synaptic plasticity (Feng and Glebov, 2021). However, excessive glutamate release at the synapse causes intracellular Ca2+ overload, increased free radical production, and Aβ formation, leading to neuronal excitotoxicity and subsequent neuronal dysfunction and apoptosis (Simões et al., 2018; Calvo-Rodriguez et al., 2020; Goel et al., 2022). This key pathophysiological mechanism is thought to underlie the widespread necrosis and functional impairment in the brains of dementia patients (Wu et al., 2021; Zhang et al., 2022). In contrast, memantine is a noncompetitive NMDA receptor antagonist that prevents NMDA overactivation and glutamate-mediated neurotoxicity, thereby protecting neuronal cells (Chayrov et al., 2022; Turcu et al., 2022). Basic research and clinical studies have shown that the combination of donepezil and memantine can address both pathologies and achieve therapeutic complementarity, resulting in more significant and cost-effective improvements in cognition and overall clinical status than either donepezil or memantine monotherapy (Yabuki et al., 2017; Guo et al., 2020; Padovani et al., 2023).

Preliminary clinical efficacy observations and safety studies have been conducted with donepezil in combination with memantine, although some adverse effects may occur in patients receiving long-term treatment (Calhoun et al., 2018; Parsons et al., 2021. However, because of the inherent limitations of clinical trials, such as the rigorous study design, strict enrollment requirements, relatively small sample sizes and short follow-up periods, and the fact that dementia usually lasts for several years, it is difficult to fully elucidate the mechanisms underlying adverse effects. For these reasons, it is difficult to fully elucidate the adverse events (AEs) of donepezil in combination with memantine for AD in the real world. More importantly, a study by Isaac et al. (2022) revealed that combination therapy increased the risk of AEs in patients compared with memantine alone. Therefore, studies based on real-world data are essential to complement the evidence on the adverse effects of donepezil in combination with memantine in the treatment of AD (Rong et al., 2023).

The U.S. FDA Adverse Event Reporting System (FAERS) database is a freely accessible database created by the FDA to collect cases of adverse drug reactions (ADRs) from around the world (Nagai and Ishikawa, 2021; Li et al., 2023). The database, which includes all AEs and medication errors recorded by the FDA, facilitates the identification and quantitative analysis of signals that indicate disproportionate reporting of ADRs, thus helping to identify correlations between specific drugs and specific AEs. Therefore, in this study, we retrieved and analyzed ADRs related to the combination of donepezil and memantine from 2004 to 2023 using the FAERS database to provide evidence and guidance for the rational and safe clinical therapeutic use of the combination of donepezil and memantine.

## 2 Materials and methods

### 2.1 Data sources

This retrospective study of ADRs was based on data from the FAERS database, a collection of reports from healthcare professionals, patients, and pharmaceutical manufacturers worldwide on ADRs, product quality complaints, and medication errors associated with marketed medicines. The data are updated quarterly and are internationally recognized for their volume and standardization. All information in the FAERS database can be downloaded free of charge (https://open.fda.gov/data/downloads/).

### 2.2 Data extraction

The data in the FAERS database include demographic and administrative information (DEMO), patient outcomes (OUTC), report sources (RPSR), coded for adverse events (REAC), therapy start and end dates for reported drugs (THER), indications for drug administration (INDI) and drug information (DRUG). Four types of drug effects were recorded in the DRUG table: primary suspected drug (PS), secondary suspected drug (SS), concomitant drug (C), and interacting drug (I) (Yang et al., 2024). Serious patient outcomes were defined as death (DE), life-threatening (LT), hospitalization-initial or prolonged (HO), disability (DS), congenital anomaly (CA), and other serious/important medical events (OT). In this study, we used the drug names in the DRUG file to identify cases and made the selection based on the results of the PS.

The AEs in the FAERS database are standardized by the Medical Dictionary for Regulatory Activities (MedDRA). The structural hierarchy of MedDRA terms is divided into five levels: system organ class (SOC), high-level group term (HLGT), high-level term (HLT), preferred term (PT), and lowest-level term (LLT). We chose the PT to code AEs to provide a structured way of summarizing and analyzing AE characteristics and ultimately mapping them to the corresponding SOC level in MedDRA. In addition, a PT can be associated with several SOCs in the MedDRA (Liu M. et al., 2024; Zou et al., 2024). Finally, drug names were standardized using the Medex UIMA 1.8.3 system.

### 2.3 Data cleaning

We extracted the data using FDA-recommended methods (Sakaeda et al., 2013). First, we downloaded the original FAERS data from January 2004 to December 2023 for the combination of donepezil and memantine. Due to the characteristics of data updating procedures, duplicate reports inevitably exist in the FAERS database, and duplicate reports may affect the reliability of disproportionate analyses (Hung et al., 2023); therefore, we deleted duplicate medical record reports using the FDA-recommended method. The method is as follows: select the PRIMARYID, CASEID, and FDA_DT fields of the DEMO table and sort them by CASEID, FDA_DT, and PRIMARYID; if there are reports with the same CASEID, keep the report with the largest FDA_DT value; if there are records with the same CASEID and FDA_DT, keep the record with the largest PRIMARYID value. If there are records with the same CASEID and FDA_DT, the record with the largest PRIMARYID value is retained (Zou et al., 2024). In addition, the specific report on the FDA website indicating the error is removed as recommended. Finally, clinical characteristics related to sex, age, reporting region, reporter, time of reporting, and outcome of AEs related to the combination of donepezil and memantine were extracted.

### 2.4 Statistical analysis

**FIGURE 1:**
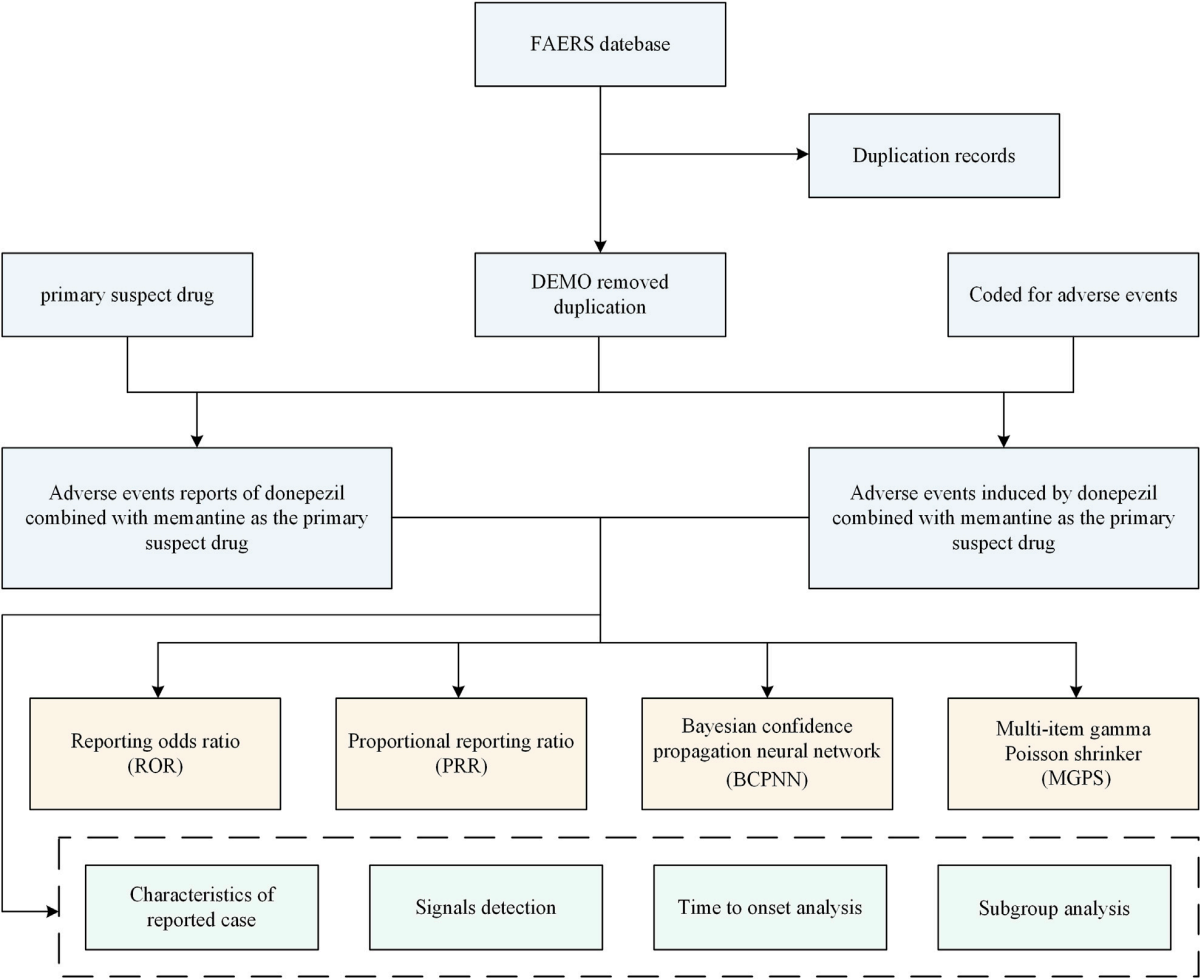
The flow diagram of selecting donepezil plus memantine therapy related AEs from the FAERS database. DEMO, demographic and administrative information; FAERS, US Food and Drug Administration Adverse Event Reporting System.

Disproportionality analysis is now widely used in the monitoring of ADRs (Bao et al., 2024). We used a four-grid proportional imbalance method for disproportionality analysis (Supplementary Table S1). Moreover, we used the commonly used in disproportionality analyses, namely, reporting odds ratio (ROR), proportional reporting ratio (PRR), Bayesian confidence propagation neural network (BCPNN), and multi-item gamma Poisson shrinker (MGPS), analyses, to analyze the summary results (Wu et al., 2024). The greater the values of the ROR, PRR, BCPNN, and MGPS were, the stronger the AE signal and the stronger the statistical relationship between the target drug and the target AE (Wu et al., 2023; Wang G. et al., 2024). The formulas of the four algorithms and the thresholds are listed in Supplementary Table S2. All of the above analyses were performed using R (version 4.2.2). The data processing flowchart is shown in Figure 1.

### 2.5 Time to onset analysis

First, cases with input errors (EVENT_DT earlier than START_DT), inaccurate data, or missing data were excluded. The time to onset was then calculated as the difference between EVENT_DT (date of AE occurrence) and START_DT (date of medication initiation). The median and interquartile range were used to describe the time to onset.

### 2.6 Visualization of data

Plots were generated using the ggplot2 package and GraphPad Prism 8.0.1. We used a world heatmap to visualize the data for the countries that provided reports. We also visualized the number of cases from 2004 to 2023 using a line graph. In addition, to determine whether the AE signal was the same between men and women after the combination of donepezil and memantine, we created a volcano plot with log2-transformed PRR values on the horizontal axis and -log10-transformed corrected *p* values on the vertical axis (Zou et al., 2024). When the PRR was greater than 1 and the P. adj was greater than 0.05, the AE signals differed between female and male patients. The sex and age ratio of reported cases and the number of reported cases per year were processed and finally plotted using Excel tables.

### 2.7 Ethics statement

Ethical review and approval were not required for this study of human participants, but local legislation and institutional requirements were followed. The national law and institutional requirement to obtain written informed consent from patients or their legal guardian/next of kin were waived for this study.

## 3 Results

### 3.1 Characteristics of the reported cases

**FIGURE 2:**
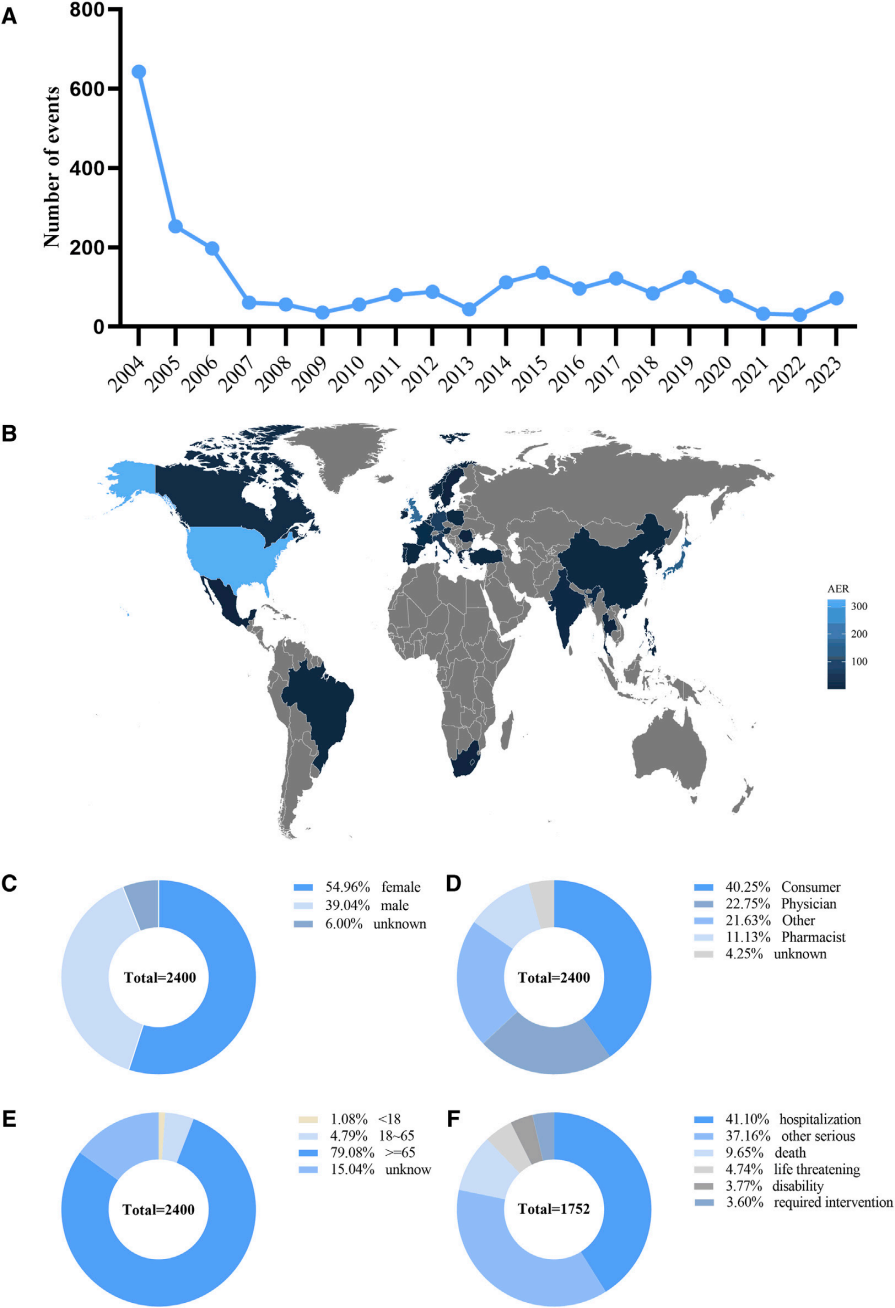
Basic information and patient characteristics according to the reports. **(A)** The annual distribution of donepezil combined with memantine administration related AEs reports from 2004 to 2023. **(B)** Country distribution of adverse events for donepezil combined with memantine administration, Darker colors represent a higher number of reports. **(C)** Gender ratio of male and female in reported events. **(D)** Occupational information ratio in reported events. **(E)** Age distribution ratio in reported events. **(F)** Ratio of outcomes in reported events. Visualization through proportional area map. Larger areas represent more reporters.

Between 2004 and 2023, 15,117,477 ADRs were reported, of which 2,400 were ADRs for the combination of donepezil and memantine. Supplementary Table S3 shows the general characteristics of the reports, including the sex and age of the patient, the year of the report, the occupation of the person making the report, and the country in which the report was made. The year 2004 was the year of the peak in the number of ADR reports for the combination of donepezil and memantine (643, 26.79%), and the number of ADR reports began to decrease after 2004 and then remained essentially stable (Figure 2A). Reporting country information was not available for 25% of all ADR reports, limiting our insight into the relationship between geographical location and AEs. However, of the reports with explicit geographic location information, the top three countries in terms of the number of reports submitted were the United States (n = 325), the United Kingdom (n = 168), and Japan (n = 155) (Figure 2B). Among all ADR reports (Figure 2C), 54.96% involved female patients, and 39.04% involved male patients, with the sex of 6% of patients in ADR reports remaining unknown. Figure 2D shows that the main sources of reports were drug consumers (40.25%) and healthcare professionals (22.75%). In terms of age (Figure 2E), the majority of ADR reports involved patients older than 65 years (79.08%). A total of 1.08% of patients were under 18 years of age, and 4.79% were between 18 and 65 years of age. Overall, 41.1% of the patients were hospitalized (Figure 2F).

### 3.2 Signal detection based on SOC levels

The signals detected at the SOC level for the combination of donepezil and memantine are shown in Table 1. Our statistical analyses showed that a total of 22 SOCs were affected by AEs associated with the combination of donepezil and memantine. When we selected SOCs that met the four index criteria and sorted them in descending order by the ROR, only psychiatric disorders (ROR = 3.57, PRR = 3.08, χ2 = 2,102.48, IC = 1.62, EBGM = 3.08) and nervous system disorders (ROR = 2.5, PRR = 3.08, IC = 1.62, EBGM = 3.08) were found to be affected. SOCs sorted in descending order according to the number of cases and a number of cases greater than 100 were nervous system disorders (n = 1,612), psychiatric disorders (n = 1,405), general disorders, and administration site conditions (n = 805), investigations (n = 700), gastrointestinal disorders (n = 510), injury, poisoning and procedural complications (n = 452) and cardiac disorders (n = 327), infections and infestations (n = 230), metabolism and nutrition disorders (n = 220), respiratory, thoracic and mediastinal disorders (n = 206), musculoskeletal and connective tissue diseases (n = 198), musculoskeletal and connective tissue disorders (n = 185), renal and urinary disorders (n = 171), and vascular disorders (n = 154).

**TABLE 1:** Signal strength of reports of donepezil plus memantine administration at the system organ class level in the FAERS database.

System organ class	Case reports	ROR (95% CI)	PRR (95% CI)	chisq	IC (IC025)	EBGM (EBGM05)
Nervous system disorders	1,612	2.78 (2.63,2.93)	2.39 (2.3, 2.49)	1,433.24	1.26	2.39
Psychiatric disorders	1,405	3.57 (3.37,3.78)	3.08 (2.96, 3.2)	2,102.48	1.62	3.08
General disorders and administration site conditions	805	0.55 (0.51,0.59)	0.6 (0.57, 0.64)	263.91	−0.74	0.6
Investigations	700	1.48 (1.37, 1.6)	1.43 (1.32,1.55)	97.7	0.52	1.43
Gastrointestinal disorders	510	0.74 (0.68,0.81)	0.76 (0.7, 0.82)	42.9	−0.4	0.76
Injury, poisoning and procedural complications	452	0.65 (0.59,0.72)	0.67 (0.61,0.74)	78.44	−0.57	0.67
Cardiac disorders	327	1.54 (1.38,1.72)	1.51 (1.37,1.67)	58.65	0.6	1.51
Infections and infestations	230	0.56 (0.49,0.64)	0.57 (0.51,0.64)	77.92	−0.81	0.57
Metabolism and nutrition disorders	220	1.31 (1.14,1.49)	1.3 (1.13, 1.49)	15.36	0.38	1.3
Respiratory, thoracic and mediastinal disorders	206	0.54 (0.47,0.62)	0.55 (0.48,0.63)	79.58	−0.86	0.55
Musculoskeletal and connective tissue disorders	198	0.46 (0.4, 0.53)	0.48 (0.42,0.55)	121.02	−1.07	0.48
Skin and subcutaneous tissue disorders	185	0.43 (0.37,0.49)	0.44 (0.38, 0.5)	138.1	−1.18	0.44
Renal and urinary disorders	171	1.17 (1.01,1.36)	1.17 (1, 1.37)	4.13	0.22	1.17
Vascular disorders	154	0.89 (0.76,1.04)	0.89 (0.76,1.04)	2.03	−0.16	0.89
Eye disorders	63	0.4 (0.31, 0.51)	0.41 (0.32,0.53)	55.84	−1.3	0.41
Hepatobiliary disorders	58	0.81 (0.62,1.05)	0.81 (0.63,1.05)	2.63	−0.3	0.81
Blood and lymphatic system disorders	47	0.35 (0.27,0.47)	0.36 (0.27,0.48)	55.01	−1.48	0.36
Endocrine disorders	23	1.18 (0.78,1.77)	1.18 (0.78,1.78)	0.61	0.23	1.18
Ear and labyrinth disorders	22	0.65 (0.43,0.98)	0.65 (0.43,0.98)	4.23	−0.63	0.65
Neoplasms benign, malignant and unspecified (incl cysts and polyps)	15	0.08 (0.05,0.14)	0.09 (0.05,0.15)	150.4	−3.55	0.09
Reproductive system and breast disorders	12	0.18 (0.1, 0.31)	0.18 (0.1, 0.32)	45.52	−2.48	0.18
Immune system disorders	5	0.06 (0.02,0.14)	0.06 (0.02,0.14)	77.16	−4.1	0.06

Abbreviations: ROR, reporting odds ratio; CI, confidence interval; PRR, proportional reporting ratio; chisq, chi-squared; IC, information component; EBGM, empirical Bayesian geometric mean; IC025, the lower limit of 95% CI, of the IC; EBGM05, the lower limit of 95% CI, of EBGM.

We found that some of the results were the same as the SOCs corresponding to common adverse reactions in the package leaflets, indicating a high level of confidence in the data. Some of the SOCs associated with significant adverse reactions, including psychiatric disorders (n = 1,405, ROR = 3.57, PRR = 3.08, χ2 = 2,102.48, IC = 1.62, EBGM = 3.08), general disorders and administration site conditions (n = 805, n = 1,219, ROR = 1.09, PRR = 3.08, χ2 = 211, IC = 1.62, EBGM = 3.08), investigations (n = 700, ROR = 1.48, PRR = 1.43, χ2 = 97.7, IC = 0.52, EBGM = 1.43), metabolism and nutrition disorders (n = 220, ROR = 1.31, PRR = 1.31, χ2 = 15.36, IC = 0.38, EBGM = 1.3), infections and infestations (n = 230, ROR = 0.56, PRR = 0.57, χ2 = 77.92, IC = −0.81, EBGM = 0.57), musculoskeletal and connective tissue disorders (n = 198, ROR = 0.46, PRR = 0.48, χ2 = 121.02, IC = −1.07, EBGM = 0.48) and vascular disorders (n = 154, ROR = 0.89, PRR = 0.89, χ2 = 2.03, IC = −0.16, EBGM = 0.89), were new and valuable adverse reactions not listed for the combination of donepezil and memantine. The remaining SOCs did not show positive results at any of the four signal intensities, except for psychiatric disorders, but because of the large number of reports of psychiatric disorders, further attention and research may be needed.

### 3.3 Signal detection based on PT levels

A PT is a detailed description of the specific clinical presentation, site of occurrence, and disease subtype of a disease or AE and is the recommended terminology for pharmacovigilance data analysis (Wang H. et al., 2024). For PT-related AEs, we selected a total of 166 PTs that met all four screening criteria, as shown in Table 2.

**TABLE 2:** Signal strength of reports of donepezil plus memantine administration at the preferred term level in the FAERS database.

PTs and categories	Case reports	ROR (95% CI)	PRR (95% CI)	chisq	IC (IC025)	EBGM (EBGM05)
Nervous system disorders						
Dizziness	182	2.82 (2.43, 3.26)	2.77 (2.41, 3.18)	208.02	1.47 (1.26)	2.77 (2.45)
Somnolence	165	6.36 (5.45, 7.42)	6.24 (5.33, 7.3)	727.26	2.64 (2.42)	6.23 (5.48)
Tremor	78	3.49 (2.79, 4.36)	3.46 (2.79, 4.29)	136.79	1.79 (1.47)	3.46 (2.87)
Syncope	60	4.48 (3.48, 5.78)	4.46 (3.46, 5.75)	161	2.15 (1.79)	4.45 (3.6)
Lethargy	59	7.59 (5.88, 9.81)	7.54 (5.84, 9.73)	334.64	2.91 (2.55)	7.53 (6.08)
Loss of consciousness	52	3.03 (2.31, 3.98)	3.02 (2.3, 3.97)	70.24	1.59 (1.2)	3.02 (2.4)
Dyskinesia	49	9.02 (6.81, 11.95)	8.97 (6.82, 11.8)	346.62	3.16 (2.76)	8.96 (7.08)
Hypersomnia	41	11.07 (8.14, 15.05)	11.01 (8.05, 15.07)	372.78	3.46 (3.02)	11 (8.5)
Speech disorder	37	5.24 (3.79, 7.24)	5.22 (3.81, 7.14)	126.13	2.38 (1.92)	5.21 (3.98)
Depressed level of consciousness	36	6.87 (4.95, 9.53)	6.84 (4.9, 9.54)	179.42	2.77 (2.31)	6.83 (5.19)
Pleurothotonus	36	290.8 (207.97,406.64)	289.4 (207.39,403.84)	9,867.91	8.11 (7.63)	276.05 (208.53)
Myoclonus	32	20 (14.12, 28.32)	19.92 (14, 28.35)	573.13	4.31 (3.82)	19.85 (14.84)
Altered state of consciousness	31	11.69 (8.21, 16.64)	11.64 (8.18, 16.56)	301.11	3.54 (3.04)	11.62 (8.65)
Encephalopathy	28	8.84 (6.1, 12.82)	8.81 (6.07, 12.79)	193.76	3.14 (2.61)	8.8 (6.45)
Psychomotor hyperactivity	28	11.92 (8.22, 17.28)	11.88 (8.19, 17.24)	278.43	3.57 (3.04)	11.85 (8.69)
Dementia	27	8.11 (5.56, 11.84)	8.09 (5.57, 11.74)	167.59	3.01 (2.48)	8.08 (5.89)
Sedation	26	8.31 (5.65, 12.22)	8.29 (5.6, 12.27)	166.45	3.05 (2.5)	8.28 (6)
Dementia alzheimer’s type	26	21.39 (14.55, 31.47)	21.32 (14.41, 31.55)	501.92	4.41 (3.86)	21.25 (15.39)
Cognitive disorder	21	3.49 (2.28, 5.36)	3.49 (2.27, 5.37)	37.23	1.8 (1.2)	3.48 (2.43)
Tardive dyskinesia	20	4.67 (3.01, 7.24)	4.66 (3.03, 7.17)	57.45	2.22 (1.6)	4.66 (3.22)
Epilepsy	19	5.02 (3.2, 7.87)	5.01 (3.19, 7.86)	60.91	2.32 (1.69)	5 (3.43)
Mental impairment	18	5.57 (3.51, 8.84)	5.56 (3.47, 8.9)	67.23	2.47 (1.82)	5.55 (3.77)
Aphasia	17	4.14 (2.57, 6.67)	4.14 (2.59, 6.63)	40.42	2.05 (1.38)	4.13 (2.78)
Extrapyramidal disorder	17	4.47 (2.78, 7.2)	4.46 (2.79, 7.14)	45.67	2.16 (1.49)	4.46 (3)
Serotonin syndrome	15	6.4 (3.86, 10.63)	6.39 (3.84, 10.64)	68.17	2.67 (1.97)	6.39 (4.18)
Cerebral atrophy	14	24.45 (14.46, 41.34)	24.4 (14.37, 41.42)	312.96	4.6 (3.87)	24.31 (15.66)
Dystonia	14	4.99 (2.95, 8.43)	4.98 (2.93, 8.45)	44.55	2.32 (1.58)	4.98 (3.21)
Generalised tonic-clonic seizure	13	6.68 (3.87, 11.51)	6.67 (3.85, 11.55)	62.59	2.74 (1.98)	6.66 (4.22)
Parkinsonism	7	5.81 (2.77, 12.21)	5.81 (2.76, 12.24)	27.86	2.54 (1.54)	5.81 (3.12)
Facial spasm	7	45.38 (21.56, 95.49)	45.34 (21.53, 95.49)	301.22	5.49 (4.49)	45 (24.15)
Drooling	6	6.95 (3.12, 15.49)	6.95 (3.11, 15.52)	30.53	2.8 (1.73)	6.94 (3.55)
Non-24-h sleep-wake disorder	6	385.14 (168.63,879.62)	384.83 (168.95,876.56)	2,157.78	8.5 (7.39)	361.57 (181.16)
Partial seizures	5	7.67 (3.19, 18.44)	7.66 (3.17, 18.5)	28.93	2.94 (1.78)	7.65 (3.67)
Incoherent	5	5.43 (2.26, 13.05)	5.42 (2.24, 13.09)	18.03	2.44 (1.28)	5.42 (2.6)
Cerebral ischaemia	5	6.57 (2.73, 15.79)	6.56 (2.72, 15.85)	23.55	2.71 (1.56)	6.56 (3.15)
Lacunar infarction	4	11.22 (4.21, 29.94)	11.22 (4.21, 29.9)	37.16	3.49 (2.22)	11.2 (4.93)
Bradykinesia	4	6.31 (2.37, 16.83)	6.31 (2.37, 16.81)	17.84	2.66 (1.39)	6.3 (2.77)
Muscle contractions involuntary	3	6.04 (1.94, 18.73)	6.03 (1.93, 18.79)	12.59	2.59 (1.18)	6.03 (2.34)
Normal pressure hydrocephalus	3	54.74 (17.56, 170.66)	54.72 (17.56, 170.55)	156.79	5.76 (4.34)	54.23 (20.95)
Circadian rhythm sleep disorder	3	28.55 (9.18, 88.79)	28.54 (9.16, 88.95)	79.35	4.83 (3.41)	28.41 (10.99)
Psychiatric disorders						
Confusional state	298	14.3 (12.74, 16.06)	13.77 (12.24, 15.49)	3,530.93	3.78 (3.61)	13.74 (12.47)
Agitation	142	14.09 (11.93, 16.64)	13.84 (11.83, 16.19)	1,690.22	3.79 (3.55)	13.81 (12.02)
Aggression	88	12.62 (10.22, 15.57)	12.48 (10.06, 15.48)	928.31	3.64 (3.34)	12.46 (10.45)
Hallucination	60	6.34 (4.91, 8.17)	6.29 (4.88, 8.12)	267.13	2.65 (2.29)	6.29 (5.08)
Abnormal behaviour	50	8.56 (6.48, 11.3)	8.51 (6.47, 11.2)	331	3.09 (2.69)	8.5 (6.73)
Hallucination, visual	42	16.24 (11.99, 22)	16.15 (12.04, 21.67)	595.68	4.01 (3.58)	16.11 (12.5)
Disorientation	40	7.2 (5.28, 9.83)	7.17 (5.24, 9.81)	212.25	2.84 (2.4)	7.16 (5.52)
Delirium	38	8.71 (6.33, 11.99)	8.67 (6.34, 11.86)	257.75	3.11 (2.66)	8.66 (6.63)
Restlessness	34	6.86 (4.9, 9.61)	6.84 (4.9, 9.54)	169.3	2.77 (2.29)	6.83 (5.15)
Irritability	25	2.98 (2.01, 4.41)	2.97 (2.01, 4.4)	32.66	1.57 (1.01)	2.97 (2.14)
Nightmare	24	5.03 (3.37, 7.5)	5.01 (3.39, 7.41)	77.09	2.32 (1.76)	5.01 (3.58)
Anger	20	4.18 (2.7, 6.49)	4.17 (2.71, 6.42)	48.26	2.06 (1.44)	4.17 (2.89)
Mania	20	8.89 (5.73, 13.79)	8.87 (5.76, 13.65)	139.42	3.15 (2.53)	8.85 (6.13)
Mental status changes	17	4.49 (2.79, 7.23)	4.48 (2.8, 7.17)	45.95	2.16 (1.5)	4.48 (3.01)
Hallucination, auditory	17	8.13 (5.05, 13.09)	8.12 (5.07, 13)	105.95	3.02 (2.35)	8.11 (5.44)
Eating disorder	16	5.66 (3.46, 9.24)	5.65 (3.46, 9.22)	61.18	2.5 (1.81)	5.64 (3.74)
Delusion	15	7.44 (4.48, 12.35)	7.43 (4.46, 12.37)	83.36	2.89 (2.18)	7.42 (4.86)
Abnormal dreams	15	3.72 (2.24, 6.17)	3.71 (2.23, 6.18)	29.72	1.89 (1.18)	3.71 (2.43)
Sopor	14	7.42 (4.39, 12.53)	7.4 (4.36, 12.56)	77.46	2.89 (2.16)	7.4 (4.77)
Paranoia	13	5.44 (3.16, 9.38)	5.43 (3.14, 9.4)	46.99	2.44 (1.68)	5.43 (3.44)
Personality change	10	7.45 (4, 13.85)	7.44 (3.97, 13.93)	55.67	2.89 (2.04)	7.43 (4.42)
Apathy	9	4.69 (2.44, 9.02)	4.69 (2.46, 8.96)	26.08	2.23 (1.33)	4.68 (2.71)
Euphoric mood	9	6.24 (3.25, 12)	6.24 (3.27, 11.91)	39.53	2.64 (1.74)	6.23 (3.6)
Flight of ideas	8	98.7 (49.06, 198.56)	98.59 (48.68, 199.65)	760.26	6.6 (5.65)	97.01 (54.05)
Behaviour disorder	8	17.16 (8.57, 34.37)	17.15 (8.64, 34.06)	121.3	4.1 (3.15)	17.1 (9.57)
Logorrhoea	7	13.77 (6.56, 28.92)	13.76 (6.53, 28.98)	82.63	3.78 (2.78)	13.73 (7.38)
Tension	6	7 (3.14, 15.6)	7 (3.13, 15.63)	30.82	2.81 (1.74)	6.99 (3.58)
Staring	5	14.66 (6.09, 35.26)	14.65 (6.06, 35.39)	63.43	3.87 (2.71)	14.61 (7.01)
Lack of spontaneous speech	5	79.58 (32.92, 192.38)	79.53 (32.92, 192.12)	382.62	6.29 (5.13)	78.5 (37.51)
Emotional poverty	5	24.3 (10.09, 58.51)	24.29 (10.05, 58.68)	111.19	4.6 (3.44)	24.19 (11.6)
Enuresis	4	7.62 (2.86, 20.31)	7.61 (2.86, 20.28)	22.95	2.93 (1.66)	7.6 (3.35)
Sleep talking	4	17.71 (6.63, 47.27)	17.7 (6.64, 47.16)	62.84	4.14 (2.87)	17.65 (7.76)
Listless	4	8.95 (3.36, 23.87)	8.95 (3.36, 23.85)	28.19	3.16 (1.89)	8.93 (3.93)
Catatonia	4	6.12 (2.3, 16.32)	6.12 (2.3, 16.31)	17.12	2.61 (1.35)	6.11 (2.69)
Abulia	4	34.55 (12.93, 92.33)	34.53 (12.96, 92)	129.48	5.1 (3.83)	34.34 (15.08)
Impulsive behaviour	4	7.85 (2.94, 20.94)	7.85 (2.95, 20.92)	23.87	2.97 (1.7)	7.84 (3.45)
Belligerence	3	45.21 (14.51, 140.8)	45.19 (14.5, 140.85)	128.66	5.49 (4.07)	44.86 (17.34)
Libido increased	3	10.46 (3.37, 32.46)	10.45 (3.35, 32.57)	25.6	3.38 (1.97)	10.44 (4.04)
Sexually inappropriate behaviour	3	55.08 (17.67, 171.72)	55.06 (17.67, 171.61)	157.78	5.77 (4.35)	54.57 (21.07)
Investigations						
Heart rate decreased	21	4.51 (2.94, 6.93)	4.5 (2.92, 6.93)	57.22	2.17 (1.57)	4.5 (3.14)
Electrocardiogram qt prolonged	18	3.91 (2.46, 6.21)	3.9 (2.44, 6.24)	38.87	1.96 (1.31)	3.9 (2.65)
Blood urea increased	17	6.87 (4.27, 11.06)	6.86 (4.29, 10.98)	85.01	2.78 (2.11)	6.85 (4.6)
Respiratory rate increased	17	14.44 (8.97, 23.26)	14.41 (9, 23.07)	211.74	3.85 (3.18)	14.38 (9.65)
Electrocardiogram pr prolongation	16	172.95 (105.16,284.45)	172.58 (105.73,281.71)	2,652.68	7.39 (6.69)	167.76 (110.63)
Blood pressure systolic increased	15	5.99 (3.61, 9.95)	5.98 (3.59, 9.95)	62.2	2.58 (1.87)	5.98 (3.91)
Blood creatine phosphokinase increased	14	3.34 (1.98, 5.64)	3.33 (1.96, 5.65)	22.89	1.74 (1.01)	3.33 (2.15)
Haematocrit decreased	12	4.13 (2.35, 7.28)	4.13 (2.34, 7.29)	28.44	2.04 (1.26)	4.13 (2.57)
Blood albumin decreased	10	9.12 (4.91, 16.97)	9.11 (4.87, 17.06)	72.13	3.19 (2.33)	9.1 (5.41)
Blood sodium decreased	9	3.65 (1.9, 7.01)	3.64 (1.91, 6.95)	17.24	1.86 (0.97)	3.64 (2.11)
Electroencephalogram abnormal	8	17.5 (8.74, 35.04)	17.48 (8.8, 34.71)	123.94	4.12 (3.18)	17.43 (9.75)
Body temperature decreased	6	4.36 (1.96, 9.71)	4.36 (1.95, 9.74)	15.5	2.12 (1.05)	4.35 (2.23)
Lipase increased	6	5.78 (2.6, 12.88)	5.78 (2.59, 12.91)	23.69	2.53 (1.46)	5.77 (2.95)
Protein total decreased	5	9.71 (4.04, 23.36)	9.71 (4.02, 23.46)	38.98	3.28 (2.12)	9.69 (4.65)
Creatinine renal clearance decreased	5	8.86 (3.68, 21.3)	8.85 (3.66, 21.38)	34.77	3.14 (1.99)	8.84 (4.24)
Blood pressure diastolic decreased	5	5.1 (2.12, 12.27)	5.1 (2.11, 12.32)	16.47	2.35 (1.19)	5.1 (2.45)
Blood chloride increased	4	17.8 (6.67, 47.51)	17.79 (6.68, 47.4)	63.21	4.15 (2.88)	17.74 (7.8)
Blood sodium increased	4	10.85 (4.07, 28.93)	10.84 (4.07, 28.88)	35.67	3.44 (2.17)	10.82 (4.76)
Computerised tomogram abnormal	3	9 (2.9, 27.94)	9 (2.89, 28.05)	21.29	3.17 (1.75)	8.98 (3.48)
Blood magnesium increased	3	24 (7.72, 74.59)	23.99 (7.7, 74.77)	65.82	4.58 (3.16)	23.9 (9.25)
Fibrin d dimer increased	3	6.63 (2.14, 20.57)	6.63 (2.13, 20.66)	14.32	2.73 (1.31)	6.62 (2.57)
Blood phosphorus increased	3	8.8 (2.84, 27.32)	8.8 (2.82, 27.43)	20.7	3.14 (1.72)	8.79 (3.41)
Brain natriuretic peptide increased	3	7.51 (2.42, 23.31)	7.51 (2.41, 23.41)	16.91	2.91 (1.49)	7.5 (2.91)
Lymphocyte percentage decreased	3	15.24 (4.91,47.32)	15.23 (4.89, 47.47)	39.78	3.93 (2.51)	15.19 (5.89)
Blood chloride decreased	3	7.87 (2.54, 24.42)	7.87 (2.53, 24.53)	17.96	2.97 (1.56)	7.86 (3.05)
Cardiac disorders						
Bradycardia	81	11.35 (9.12, 14.13)	11.24 (9.06, 13.94)	754.92	3.49 (3.17)	11.22 (9.34)
Sinus bradycardia	22	16.87 (11.09, 25.65)	16.82 (11.14, 25.39)	326.52	4.07 (3.48)	16.78 (11.81)
Atrioventricular block first degree	14	22.1 (13.07, 37.36)	22.06 (13, 37.45)	280.42	4.46 (3.73)	21.98 (14.16)
Atrioventricular block complete	9	10.22 (5.31, 19.66)	10.21 (5.35, 19.5)	74.62	3.35 (2.45)	10.19 (5.89)
Ventricular fibrillation	8	5.26 (2.63, 10.53)	5.26 (2.65, 10.45)	27.57	2.39 (1.45)	5.26 (2.94)
Ventricular extrasystoles	7	4.79 (2.28, 10.05)	4.78 (2.27, 10.07)	20.93	2.26 (1.26)	4.78 (2.57)
Cardiac failure acute	6	7.47 (3.35, 16.64)	7.46 (3.34, 16.66)	33.54	2.9 (1.83)	7.45 (3.81)
Bundle branch block left	5	8.31 (3.46, 19.99)	8.31 (3.44, 20.07)	32.1	3.05 (1.9)	8.3 (3.98)
Supraventricular extrasystoles	4	8.3 (3.11, 22.14)	8.3 (3.12, 22.11)	25.64	3.05 (1.79)	8.29 (3.65)
Sinoatrial block	3	34.29 (11.02, 106.7)	34.28 (11, 106.84)	96.38	5.09 (3.67)	34.09 (13.19)
Injury, poisoning and procedural complications						
Fall	136	3.14 (2.65, 3.72)	3.1 (2.65, 3.63)	195.05	1.63(1.39)	3.1 (2.69)
Accidental overdose	23	5.11 (3.39, 7.7)	5.1 (3.38, 7.7)	75.73	2.35 (1.77)	5.09 (3.62)
Accidental exposure to product by child	16	17.69 (10.82, 28.91)	17.65 (10.81, 28.81)	250.66	4.14 (3.45)	17.6 (11.67)
Product administration interrupted	8	13.26 (6.62, 26.54)	13.24 (6.67, 26.29)	90.36	3.72 (2.78)	13.22 (7.39)
Skin laceration	7	5.7 (2.72, 11.97)	5.7 (2.71, 12)	27.09	2.51 (1.51)	5.69 (3.06)
Femoral neck fracture	5	7.34 (3.05, 17.65)	7.34 (3.04, 17.73)	27.33	2.87 (1.72)	7.33 (3.52)
Skin abrasion	4	7.19 (2.7, 19.17)	7.19 (2.7, 19.16)	21.28	2.84 (1.58)	7.18 (3.16)
Wrong patient received product	3	11.48 (3.7, 35.65)	11.48 (3.68, 35.78)	28.64	3.52 (2.1)	11.46 (4.44)
Jaw fracture	3	9.8 (3.16, 30.42)	9.79 (3.14, 30.51)	23.65	3.29 (1.87)	9.78 (3.79)
Metabolism and nutrition disorders						
Hypernatraemia	16	24.97 (15.27, 40.82)	24.92 (15.27, 40.68)	365.84	4.63 (3.95)	24.82 (16.45)
Hypophagia	12	3.75 (2.13, 6.6)	3.74 (2.12, 6.6)	24.09	1.9 (1.12)	3.74 (2.33)
Failure to thrive	7	9.45 (4.5, 19.83)	9.44 (4.48, 19.88)	52.73	3.24 (2.24)	9.42 (5.07)
Hypovolaemia	6	7.54 (3.39, 16.8)	7.54 (3.38, 16.84)	33.97	2.91 (1.84)	7.53 (3.85)
Hyperphagia	6	12.19 (5.47, 27.17)	12.18 (5.45, 27.2)	61.46	3.6 (2.53)	12.16 (6.22)
Hyperglycaemic hyperosmolar nonketotic syndrome	5	41.86 (17.36, 100.9)	41.83 (17.32, 101.05)	197.88	5.38 (4.22)	41.54 (19.9)
Polydipsia	5	9.05 (3.76, 21.75)	9.04 (3.74, 21.84)	35.7	3.17 (2.02)	9.03 (4.33)
Diet refusal	4	35 (13.1, 93.56)	34.98 (13.13, 93.2)	131.28	5.12 (3.85)	34.79 (15.28)
General disorders and administration site conditions						
Drug interaction	62	2.95 (2.3, 3.79)	2.94 (2.28, 3.79)	79.38	1.55 (1.2)	2.94 (2.38)
Crying	16	3.07 (1.88, 5.02)	3.07 (1.88, 5.01)	22.31	1.62 (0.93)	3.07 (2.03)
Hypothermia	15	11.06 (6.66, 18.37)	11.04 (6.63, 18.38)	136.8	3.46 (2.75)	11.03 (7.21)
Screaming	8	9.31 (4.65, 18.64)	9.3 (4.68, 18.47)	59.2	3.22 (2.27)	9.29 (5.2)
Foaming at mouth	7	25.44 (12.1, 53.46)	25.41 (12.07, 53.51)	163.48	4.66 (3.66)	25.31 (13.6)
Sluggishness	6	4.44 (1.99, 9.88)	4.43 (1.98, 9.89)	15.95	2.15 (1.08)	4.43 (2.27)
Energy increased	4	4.84 (1.81, 12.89)	4.83 (1.81, 12.87)	12.15	2.27 (1.01)	4.83 (2.13)
Infections and infestations						
Urinary tract infection	58	2.69 (2.08, 3.48)	2.68 (2.08, 3.46)	61.14	1.42 (1.05)	2.68 (2.16)
Pneumonia aspiration	17	5.24 (3.25, 8.43)	5.23 (3.27, 8.37)	58.08	2.38 (1.72)	5.22 (3.51)
Gastrointestinal infection	11	9.78 (5.41, 17.67)	9.76 (5.42, 17.57)	86.4	3.29 (2.47)	9.75 (5.94)
Urosepsis	6	5 (2.25, 11.14)	5 (2.24, 11.17)	19.18	2.32 (1.25)	5 (2.56)
Sialoadenitis	3	17.5 (5.63, 54.36)	17.49 (5.61, 54.51)	46.51	4.12 (2.71)	17.44 (6.76)
Renal and urinary disorders						
Urinary incontinence	43	10.81 (8.01, 14.6)	10.76 (8.02, 14.44)	380.06	3.42 (3)	10.74 (8.36)
Pollakiuria	22	4.02 (2.65, 6.11)	4.01 (2.66, 6.05)	49.8	2 (1.41)	4.01 (2.83)
Hydronephrosis	6	6.07 (2.73, 13.53)	6.07 (2.72, 13.56)	25.39	2.6 (1.53)	6.06 (3.1)
Ketonuria	5	44.88 (18.61, 108.21)	44.85 (18.57, 108.35)	212.76	5.48 (4.32)	44.52 (21.32)
Gastrointestinal disorders						
Constipation	67	2.53 (1.99, 3.21)	2.51 (1.98, 3.18)	61.23	1.33 (0.98)	2.51 (2.05)
Salivary hypersecretion	13	10.06 (5.83, 17.34)	10.04 (5.8, 17.38)	105.68	3.33 (2.57)	10.03 (6.36)
Eructation	12	5.6 (3.18, 9.86)	5.59 (3.17, 9.87)	45.19	2.48 (1.7)	5.58 (3.48)
Faecaloma	6	8.82 (3.96, 19.64)	8.81 (3.94, 19.68)	41.49	3.14 (2.07)	8.8 (4.5)
Vascular disorders						
Circulatory collapse	13	5.38 (3.12, 9.27)	5.37 (3.1, 9.3)	46.24	2.42 (1.67)	5.37 (3.4)
Hypertensive crisis	6	3.97 (1.78, 8.84)	3.97 (1.78, 8.87)	13.31	1.99 (0.92)	3.97 (2.03)
Thrombophlebitis	3	5.94 (1.91, 18.44)	5.94 (1.91, 18.51)	12.31	2.57 (1.15)	5.93 (2.3)
Skin and subcutaneous tissue disorders						
Skin oedema	9	64.99 (33.68, 125.4)	64.91 (33.99, 123.94)	560.27	6.01 (5.1)	64.23 (37.06)
Yellow skin	6	9.38 (4.21, 20.89)	9.37 (4.2, 20.93)	44.8	3.23 (2.16)	9.36 (4.79)
Decubitus ulcer	5	4.46 (1.85, 10.71)	4.45 (1.84, 10.75)	13.38	2.15 (1)	4.45 (2.14)
Musculoskeletal and connective tissue disorders						
Rhabdomyolysis	25	4.53 (3.06, 6.71)	4.52 (3.05, 6.69)	68.44	2.17 (1.62)	4.51 (3.25)
Posture abnormal	4	9.05 (3.39, 24.13)	9.04 (3.39, 24.09)	28.57	3.17 (1.91)	9.03 (3.97)
Torticollis	3	10.59 (3.41, 32.88)	10.59 (3.4, 33.01)	26	3.4 (1.99)	10.57 (4.1)
Eye disorders						
Miosis	10	10.25 (5.51, 19.06)	10.23 (5.46, 19.15)	83.19	3.35 (2.5)	10.22 (6.08)
Eye movement disorder	6	6.57 (2.95, 14.63)	6.56 (2.94, 14.65)	28.26	2.71 (1.64)	6.56 (3.35)
Respiratory, thoracic and mediastinal disorders						
Hiccups	11	10.59 (5.86, 19.14)	10.58 (5.88, 19.05)	95.22	3.4 (2.58)	10.56 (6.43)
Reproductive system and breast disorders						
Prostatomegaly	5	9.68 (4.02, 23.28)	9.67 (4, 23.36)	38.83	3.27 (2.12)	9.66 (4.64)
Hepatobiliary disorders						
Drug-induced liver injury	13	4.12 (2.39, 7.1)	4.11 (2.37, 7.12)	30.65	2.04 (1.28)	4.11 (2.61)
Endocrine disorders						
Inappropriate antidiuretic hormone secretion	17	13.13 (8.15, 21.14)	13.1 (8.18, 20.97)	189.58	3.71 (3.04)	13.07 (8.77)

Abbreviations: ROR, reporting odds ratio; CI, confidence interval; PRR, proportional reporting ratio; chisq, chi-squared; IC, information component; EBGM, empirical Bayesian geometric mean; IC025, the lower limit of 95% CI, of the IC; EBGM05, the lower limit of 95% CI, of EBGM; PTs: preferred terms.

The number of cases was more than 100, which indicates a strong signal of AEs (Yu et al., 2024), so we ranked the PT entries in descending order by the number of cases and screened the PT entries with more than 100 cases. After excluding PTs as possible indications for the combination of donepezil and memantine and nondrug signals, the PT entries with more than 100 cases were dizziness (n = 182), somnolence (n = 165), and fall (n = 136).

As the BCPNN method is more cautious and is associated with a lower chance of misclassifying early warning signals (Du et al., 2024), we ranked these PTs in numerical descending order of BCPNN. After excluding PTs as possible indications for the combination of donepezil and memantine and nondrug-related signals, the top ten PTs for the combination of donepezil and memantine were non-24-h sleep-wake disorder (EBGM = 361.57), pleurothotonus (EBGM = 276.05), electrocardiogram PR prolongation (EBGM = 167.76), flight of ideas (EBGM = 97.01), lack of spontaneous speech (EBGM = 78.5), skin edema (EBGM = 64.23), sexually inappropriate behavior (EBGM = 54.57), normal pressure hydrocephalus (EBGM = 54.23), facial spasm (EBGM = 45) and belligerence (EBGM = 44.86).

We also considered IC values, as the Bayesian approach increases the stability of calculations in the presence of a small number of AEs (Zou et al., 2024), and despite the small number of cases, we found that non-24-h sleep-wake disorder (n = 6, IC = 8.5), pleurothotonus (n = 36, IC = 8.11), electrocardiogram PR prolongation (n = 16, IC = 7.39), flight of ideas (n = 8, IC = 6.6), lack of spontaneous speech (n = 5, IC = 6.29), skin edema (n = 9, IC = 6.01), sexually inappropriate behavior (n = 3, IC = 5.77), normal pressure hydrocephalus (n = 3, IC = 5.76), facial spasm (n = 7, IC = 5.49) and belligerence (n = 3, IC = 5.49) were unexpected signals with higher IC values, suggesting a close association with the combination of donepezil and memantine.

In conclusion, we found that dizziness and electrocardiogram PR prolongation were consistent with the warnings in the package insert and on the drug label. However, non-24-h sleep-wake disorder, pleurothotonus, lack of spontaneous speech, skin edema, normal pressure hydrocephalus, facial spasm, and belligerence were not mentioned in the package leaflet, and further investigation is warranted.

### 3.4 Time to onset analysis

**FIGURE 3:**
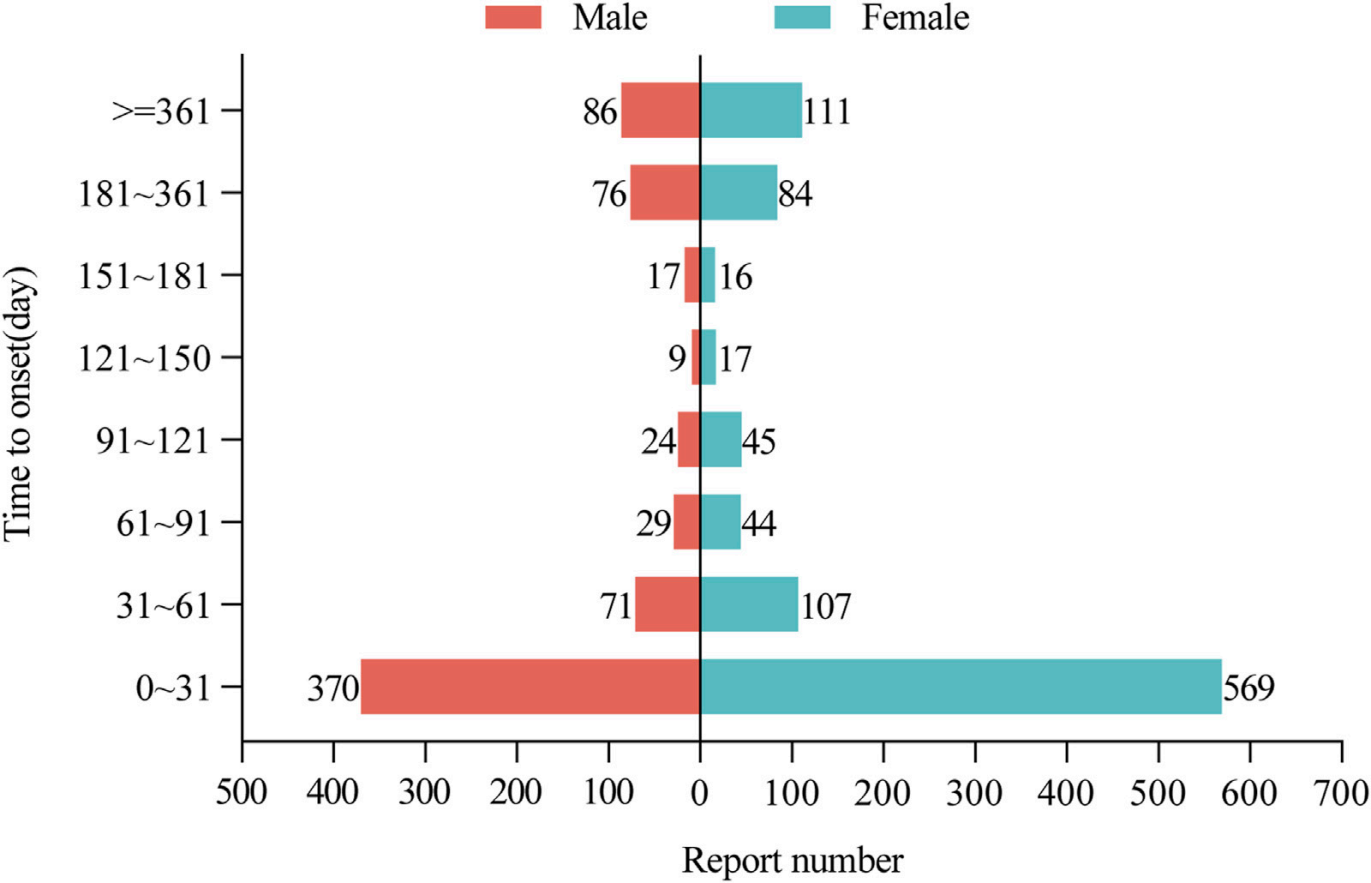
Time to onset of AEs in male and female patients receiving donepezil and memantine combination therapy. AEs: adverse events.

Of all AEs reported, a total of 2,400 reports included the time of onset of the AE, with a median time of onset of 19 days (interquartile range 3–95). After excluding reports with inaccurate, missing, or unknown sex at the time of onset, a total of 2,256 donepezil and memantine combination AE reports included the time of onset. Figure 3 shows that the time of AE onset in men (n = 569) and women (n = 370) was predominantly within 1 month of the initiation of donepezil in combination with memantine. Interestingly, AEs could still occur after 1 year of treatment with the combination of donepezil and memantine in men (n = 111) and women (n = 86). This finding also suggests the need for continued monitoring of patients for possible AEs during combination therapy with donepezil and memantine, even after 1 year of treatment.

### 3.5 Subgroup analyses

Subgroup analyses not only reduce confounding by demographic characteristics but also provide important insights for refining clinical management strategies, allowing clinical decision-makers to tailor treatment regimens to the specific characteristics of these subgroups.

#### 3.5.1 Sex in different PT groups

We analyzed whether sex affects adverse reactions to the combination of donepezil and memantine and identified 88 adverse reaction PTs in men and 100 in women using four statistical methods, the results of which are presented in Supplementary Tables S4, S5. In the case of ADRs with a case number greater than 20, we removed the indications given in the ADR reports. Somnolence, bradycardia, lethargy, dyskinesia and urinary incontinence were common to both male and female PTs; as most of the case numbers after subgroup analyses were less than 50, we used the IC values in descending order, and among PTs with more than 20 cases, bradycardia, lethargy, urinary incontinence, dyskinesia and somnolence were common to both male and female PTs.

**FIGURE 4:**
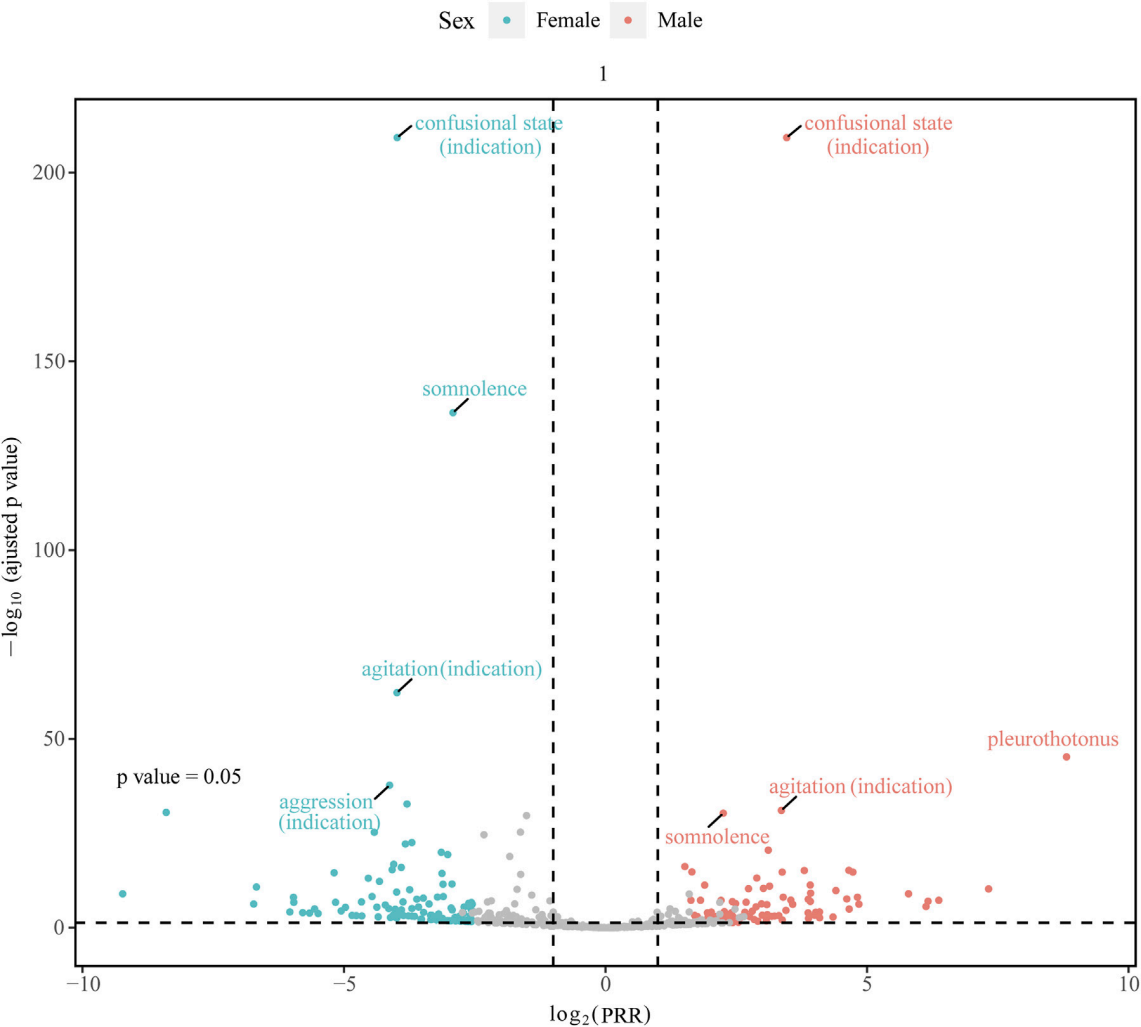
Gender-differentiated risk signal volcano plot for donepezil combined with memantine. The horizontal coordinate shows the log2 PRR value and the vertical coordinate indicates the adjusted *p*-value after -log10 conversion. PRR, proportional reporting ratio.

The “volcano plot” in Figure 4 presents the sex differences in AE signal extraction after combination therapy with donepezil and memantine. Each point in the figure represents the AE of the combination of donepezil and memantine, and we labeled the significant AEs. The blue dots indicate potential AE signals in male patients, while the red dots indicate potential AE signals in female patients. Somnolence was a more common AE in women than in men, while pleurothotonus was a separate AE in men. The above results illustrate sex-specific information on potential AE signals associated with the combination of donepezil and memantine, highlighting the differences in AEs reported in men and women and the need for separate attention in clinical management.

#### 3.5.2 Age in different PT groups

Age is an important independent risk factor for neurocognitive disorders (Ni et al., 2022). The prevalence of neurocognitive impairment increases with age (Gross et al., 2024). We performed age-stratified analyses to reduce the confounding effect of age in adverse reaction.

To analyze whether age affects adverse reactions to the combination of donepezil and memantine, we used four statistical methods to determine the PTs for 12 adverse reactions in patients aged less than 18 years, 16 in patients aged between 18 and 65 years, and 113 in patients aged more than 65 years, the results of which are presented in Supplementary Tables S6–S8. We used case numbers in descending order and excluded indications reported in the adverse reaction reports. The three most common ADRs in patients younger than 18 years were accidental overdose (n = 8), eye movement disorder (n = 5), and dystonia (n = 4). The top three PTs in patients aged 18–65 years were pleurothotonus (n = 15), myoclonus (n = 12), and disease progression (n = 6). The top three PTs in patients older than 65 years were dizziness (n = 165), somnolence (n = 153) and tremor (n = 69).

As the number of cases after subgroup analyses was mostly less than 50, we again used IC values in descending order. The three most common PTs in patients aged less than 18 years were toxic encephalopathy (IC = 6.85), eye movement disorder (IC = 6.43), and respiratory rate increase (5.83). The three most common PTs in patients aged 18–65 years were pleurothotonus (IC = 11.1), inappropriate antidiuretic hormone secretion (IC = 7.07), and myoclonus (IC = 7.03). The top three PTs in patients older than 65 years were non-24-h sleep-wake disorder (IC = 8.43), flight of ideas (IC = 7.95), and electrocardiogram PR prolongation (IC = 7.04). Finally, lethargy was a common PT in all three age groups among all PTs. The above findings suggest that different age groups have different AEs, but lethargy was the common AE among all age groups.

## 4 Discussion

To our knowledge, this is the first detailed and systematic pharmacovigilance study of relevant adverse reactions associated with donepezil in combination with memantine dosing based on the FAERS database. Our study not only highlights some of the existing safety information but also identifies new potential risks. As the number of patients with dementia increases and the clinical use of donepezil in combination with memantine is expected to expand accordingly, ongoing pharmacovigilance analysis is important not only to clarify the overall safety profile but also to provide more comprehensive and accurate data to support medical practice and public health decision-making.

The AEs of donepezil combined with memantine administration were mainly related to nervous system disorders, psychiatric disorders, general disorders, administration site conditions, and gastrointestinal disorders. Notably, for nervous system disorders and gastrointestinal disorders as well as cardiac disorders, our results provide a revalidation of these disorders from the pharmacovigilance point of view. On the other hand, psychiatric disorders, general disorders, administration site conditions, investigations, and metabolism and nutrition disorders are not mentioned in the package inserts, and further attention and investigation are warranted.

At the PT level, our study revealed that although agitation, hallucination, and confusional state were significant in the disproportionality analyses and were classified as adverse effects by some studies (Atri et al., 2013; Isaac et al., 2022), we found that they were also indications for treatment, as shown in Table 1 and other studies (Levy et al., 2012; Di Santo et al., 2013). Furthermore, 90% of dementia patients experience at least one of the behavioral and psychiatric symptoms of agitation, hallucinations, and confusional state over the course of their disease (Mitchell et al., 2016; Joshi et al., 2022). Therefore, for the accuracy of the study results, we excluded the adverse reactions reported in Table 1 from our results.

Dizziness and electrocardiogram PR prolongation are mentioned in the leaflet. Dizziness is caused by the overactivation of nicotinic receptors, and studies have shown that the incidence of dizziness is at least twice as high in the memantine plus donepezil group as in the placebo plus donepezil group (Atri et al., 2013; Grossberg et al., 2015; Cao et al., 2020). Adverse effects on cardiac function, such as arrhythmias (Kobayashi et al., 2023) and bradycardia (Babai et al., 2010), have been reported in many studies. Because of the progressive deterioration of conduction and sinus node function in elderly individuals and the high distribution of cholinesterase in the heart, cholinesterase inhibitors may affect cardiac function by increasing ACh levels via vagal effects (Kho et al., 2021).

Our study also revealed several adverse reactions, such as epilepsy and gastrointestinal adverse reactions, which were not reported in large numbers but have been reported in previous studies and are serious. Partial seizures, generalized tonic‒clonic seizures, and epilepsy may be caused by donepezil-induced metabolic disturbances leading to hyponatremic seizures (Shareef et al., 2017; Ruangritchankul et al., 2021; Ha et al., 2022). In contrast, patients with AD have a 6- to 10-fold increased risk of seizures and epilepsy compared with healthy individuals (Pandis and Scarmeas, 2012). The gastrointestinal AEs included nausea, vomiting, diarrhea, constipation, and anorexia, which were reported in the package leaflet and several studies (Atri et al., 2013; Grossberg et al., 2013; Ruangritchankul et al., 2021; Kose et al., 2023). The incidence of diarrhea was at least two times greater in the memantine plus donepezil group than in the placebo plus donepezil group (Grossberg et al., 2015). After the combination therapy was administered to 154 patients, 33 experienced gastrointestinal AEs (Cao et al., 2020). It is possible that the AEs were caused by the lower body weight of the patients, which made them less tolerant of the AEs (Han et al., 2017; Hong et al., 2019). Another reason is that donepezil inhibits the rapid hydrolysis of ACh in the peripheral nervous system, which ultimately leads to diarrhea, nausea, and vomiting (Gauthier, 2001; Brinkman et al., 2019; Ruangritchankul et al., 2021).

AEs not mentioned in the package leaflet, such as fall, pleurothotonus, and myoclonus, were also identified in this study. There were many reported fall cases in our study. The findings of several studies are consistent with our findings (Atri et al., 2013; Kose et al., 2023), in which one of the most common AEs in the combination therapy group was fall (Porsteinsson et al., 2008). Although there are currently no studies indicating the mechanism underlying this decrease, we speculate that it may be an adverse reaction of cardiac function causing cerebral ischemia that ultimately leads to a fall (Ruangritchankul et al., 2021). Pleurothotonus, myoclonus, and dystonia may be due to the overactivation of nicotinic receptors or a dopaminergic-cholinergic imbalance, which have also been reported in related studies (Zannas et al., 2014). Finally, although somnolence and lethargy are relatively less harmful and ultimately difficult to detect in the clinical setting, our results, together with those of other studies, suggest that they are among the adverse effects of donepezil administration in combination with memantine (Ovejero-Benito et al., 2022; Kose et al., 2023). Although adverse effects such as headache (Majidazar et al., 2022; Ovejero-Benito et al., 2022; Kose et al., 2023), low hemoglobin (Grossberg et al., 2013), rhabdomyolysis (Fleet et al., 2019), nasopharyngitis (Atri et al., 2013; Wong, 2016), and weakness (Babai et al., 2010) were not found in our study, they have been reported in other studies. These adverse effects are less likely to occur but still deserve our attention.

Our study revealed that female patients were more likely than male patients to report adverse reactions to donepezil in combination with memantine. We explain the phenomenon of sex differences in AEs from a sociological rather than a biological perspective. First, women live longer (Mielke et al., 2014), and the proportion of clinically diagnosed cases of dementia and AD is greater among women. A study by Tahami Monfared et al. (2022) revealed that AD affects 3.31% of men and 7.13% of women and the lifetime risk of AD dementia was estimated at 41.9% for women and 33.6% for men. In addition, improvements in women’s education and careers in recent decades may have led female patients to believe that anti-dementia drugs can lead to better physical health (Lu et al., 2021), whereas men tend to be less concerned about healthcare (Owens, 2008; Ippoliti et al., 2023). This belief also contributes to the tendency of women to report AEs more frequently and, ultimately, to more reports of ADRs in women. While the above is the sociological perspective, the biological perspective is as follows. First, it has been shown that there are differences in brain structure and function between men and women, with the hippocampus and gray matter regions being larger in women than in men (Cosgrove et al., 2007). The expression of presynaptic and postsynaptic proteins in the hippocampus is affected by fluctuations in estrogen levels, which have a direct impact on cognition (Vieira et al., 2023). Decreasing estrogen levels or menopause in women may lead to the onset or worsening of cognitive deficits and the development of dementia (Davey, 2017; Vieira et al., 2023). In addition, the ratio of the volume of distribution to the bioavailability of donepezil is greater in women than in men, and the peak concentrations of memantine are greater in women than in men (Ovejero-Benito et al., 2022). Therefore, if the same dose of donepezil and memantine is given to women and men, owing to their distribution kinetics, donepezil and memantine will have a longer period of action in women, and it will take longer for the drug to be cleared from the body in women. On the other hand, women may have lower levels of liver and kidney function than men, and there are sex differences in the activity of drug-metabolizing enzymes, all of which can affect the rate of drug metabolism (Zucker and Prendergast, 2020). As a result, women are at greater risk of experiencing adverse effects from coadministration. However, we have found in previous studies that the results of trials on whether there is a sex difference in antidementia drug response are inconsistent (Haywood and Mukaetova-Ladinska, 2006; Gallucci et al., 2016), and there are no sex-specific antidementia drug pharmacokinetic data. However, we hypothesize that women have more AEs than men due to the biological and sociological factors mentioned above, and further research is needed to investigate the underlying mechanisms and causes involved.

Our study revealed that patients over 65 years of age were more likely to report AEs with donepezil in combination with memantine than patients aged 18–65 years and patients younger than 18 years. We believe the reasons for this are as follows. (i) Most patients with AD are diagnosed after the age of 65 years. Studies have shown an AD prevalence of 11% in people over 65 years of age, and the incidence increases with age, with a prevalence of over 50% in people over 85 years of age (Hebert et al., 2013). (ii) Several bodily functions begin to decline in older patients. For example, reduced gastrointestinal motility, delayed gastric emptying time, hepatic and renal blood flow, hepatic and renal masses, and size can lead to conditions such as reduced drug absorption, prolonged drug excretion, reduced drug clearance, and increased drug blood concentration (Coin et al., 2016; Khatib et al., 2021; Ruangritchankul et al., 2021). The effects of cholinesterase inhibitors are concentration dependent, increasing the susceptibility of women, elderly individuals, and patients with chronic kidney disease to adverse effects such as the cardiac arrhythmias caused by cholinesterase inhibitors (Kho et al., 2021; Kobayashi et al., 2023). The above findings suggest that pharmacokinetic and pharmacodynamic changes due to organ aging are another cause of increased drug sensitivity and adverse reactions in elderly patients (Campbell et al., 2015; Reeve et al., 2017; Kobayashi et al., 2023). (iii) Aging and frailty in elderly individuals reduce the serum albumin concentration by 10%–20%, which plays a major role in plasma protein binding (Reeve et al., 2015). Donepezil is 75% albumin bound, a decrease in albumin binding may increase the unbound fraction with pharmacological activity, leading to greater potency and toxicity (Yamasaki et al., 2013; Yang et al., 2014; Tayyab and Feroz, 2021). In addition, donepezil may displace other highly protein-bound drugs, leading to an increase in the unbound form of these drugs and serious side effects (Tiseo et al., 1998; Ruangritchankul et al., 2021). (iv) The permeability and integrity of the blood‒brain barrier (BBB) change in older adults, the number of receptor sites may change, affecting the efficacy of many drugs (van Assema et al., 2012; Maher et al., 2021). These changes may result in increased levels of drugs crossing the BBB. The cholinergic receptors in the brain are highly sensitive, and the body’s homeostasis is reduced, which in turn becomes a predisposing factor for triggering adverse reactions and ultimately leads to the development of associated adverse effects (Mehta et al., 2015). (v) Older patients often have comorbidities and require multiple medications (Clague et al., 2017). This polypharmacy can lead to a greater risk of adverse or harmful drug reactions or drug AEs (Wastesson et al., 2018). For example, concomitant use of β-blockers, calcium channel blockers, or antiarrhythmics in people with dementia treated with acetylcholinesterase inhibitors (AChEIs) may lead to adverse cardiovascular effects such as arrhythmias, heart block, syncope and prolongation of the QT interval (Wiśniowska et al., 2016; Khatib et al., 2021; Ruangritchankul et al., 2021). On the other hand, drug-related problems such as potential drug‒drug interactions, drug-disease interactions, inappropriate medication use, and poor medication adherence can lead to adverse effects (Elliott et al., 2015; Pfister et al., 2017; Wucherer et al., 2017). Epidemiological surveys have shown that deaths from dementia have increased by more than 145% between 2000 and 2019, and the proportion of older people in the total population is expected to increase (Alzheimer’s Disease Facts and Figures, 2023). Therefore, the adverse effects of medicine combinations on elderly individuals are needed, and comprehensive medication reviews and the optimization of drug prescribing strategies are needed to address drug-related problems and ADRs.

The incidence of AEs may also increase with age because patients with advanced AD may require higher doses of medication (Hong et al., 2019). In addition, the pharmacological properties and pharmacokinetic differences of each antidementia drug may affect the accumulation and incidence of adverse effects of the drug in different patients on different regions (Noetzli and Eap, 2013; Han et al., 2017; Ovejero-Benito et al., 2022). Therefore, further experimental studies and clinical observations are essential to elucidate the mechanisms underlying the adverse effects caused by the administration of donepezil in combination with memantine. At the same time, we need to understand the adverse effects of drug combinations for scientific management. Finally, we need to minimize the effective dose, rather than adding other drugs to treat the adverse effects, to reduce the incidence of adverse outcomes, and we need to monitor patients closely after administration to achieve individualized management (Eshetie et al., 2018).

## 5 Limitations

Our study had some inherent limitations. First, (i) AEs were spontaneously reported to the FAERS, and information underreporting and overreporting, as well as inaccurate and incomplete (missing data) information; and (ii) the roles of the reporters varied. Clients, lawyers, health professionals, and nonhealthy professionals all reported cases to the FAERS. A total of 40.25% of the reports in our study were made by consumers (n = 966). (ii) The database is maintained by the US FDA, so there is inevitably a lack of cases from other countries or differences in the importance attached to AEs in different countries and regions, which may introduce bias into the analyses by restricting them to populations in a particular region. Second, only 2400 AE reports related to the combination of donepezil and memantine were extracted for this study. The sample size limitation may have caused some rare adverse reactions to be missed, and thus more studies and more reports are needed to validate our results. Third, due to the lack of a population base for the combination of donepezil and memantine, it was again impossible to calculate the incidence of adverse reactions associated with the combination of donepezil and memantine. Therefore, our findings represent only statistical correlations, and further clinical follow-up and observational and pharmacological studies are needed to determine whether a biological causal relationship exists. Despite these limitations, our results may provide ideas for further studies, and this article may serve as a valuable reference for healthcare professionals to monitor AEs associated with the combination of donepezil and memantine.

## 6 Conclusion

In this study, we observed a wide range of adverse reactions in patients who received donepezil and memantine. We observed the same adverse reactions as described in the specification, but we also found new important adverse reactions and sex and age differences in some of the adverse reactions. These findings suggest that prospective clinical trials are needed to confirm these results and to determine the relationships among them. In conclusion, this study not only provides additional information about the safety of the combination of donepezil and memantine in the clinical setting but can also help clinicians make informed decisions in clinical practice.

## Data Availability

The original contributions presented in the study are included in the article/Supplementary Material, further inquiries can be directed to the corresponding authors.
